# Cervical Cancer in Khon Kaen, Thailand: Analysis of 1990-2014 Incidence Data and Prediction of Future Trends

**DOI:** 10.31557/APJCP.2019.20.2.369

**Published:** 2019

**Authors:** Thitima Saenrueang, Supannee Promthet, Supot Kamsa-Ard, Prasit Pengsaa

**Affiliations:** 1 *Doctor of Philosophy Program in Epidemiology and Biostatistics,*; 3 *Department of Epidemiology and Biostatistics, Faculty of Public Health, *; 2 *ASEAN Cancer Epidemiology and Prevention Research Group, Khon Kaen University, Thailand.*

**Keywords:** Cervical cancer, time trend, population, based cancer registry data

## Abstract

**Background:**

Cervical cancer is the second most common cancer of women in Thailand. There have been no reports of incidence and future in Khon Kaen, a province in northeastern Thailand, where the relatively high prevalence gives evaluation of cervical cancer screening a high priority.

**Objectives:**

To determine cervical cancer incidence rates in Khon Kaen for 1990–2014 and predict future trends until 2029.

**Methods:**

Cancer incidence data from the Khon Kaen population-based cancer registry were analyzed and age-standardized incidence rates (ASR) were estimated. Joinpoint analysis and age-period-cohort modeling were applied for data from 1990 to 2014 and the Nordpred package was employed to project trends from 2015 to 2029.

**Results:**

Between 1990 and 2014, a total of 3,258 cases were diagnosed with ICD-O code C53 (invasive cervical cancer). Before 2005, an annual percentage change (APC) varied widely, with outliers in 1993 and 1999. The APC computed with the Joinpoint software decreased at -2.8% (95% CI;-4.5 to -1.1) per year on average. After 2005, a rise was noted until 2008, after which a drop became apparent with an APC of -8.0% (95% CI; -14.5 to -1.1) per year on average. Both period and cohort effects played a role in shaping the decrease in incidence. The three projection method suggested that incidence rates would continue to decrease in the future.

**Conclusions:**

A decreasing trend in incidence of cervical cancer in Khon Kaen was noted from 1990 to 2014 with a prediction of continuous decrease until 2029. Maintenance and improvement of the screening program is advised.

## Introduction

Cervical cancer is the fourth most common cancer in women worldwide, with an estimated 528,000 new cases and 266,000 deaths, accounting for 7.5% of all female cancer mortality in 2012. High-risk regions, with age-standardized incidence rates (ASR) over 30 per 100,000) include Eastern Africa, Melanesia, Southern and Middle Africa. ASRs are lowest in Australia, New Zealand and Western Asia. In addition, 87% of cervical cancer deaths occur in less developed regions (Ferlay et al., 2015).

In Thailand, in the period of 1998-2000, cervical cancer was the leading cancer in the female population. The estimated ASRs reached a peak of around 24.7 per 100,000 as documented in Cancer in Thailand volume IV (Khuhaprema et al., 2007). At this time, cervical cancer was the second most common cancer in Thai females although the estimated ASR continuously dropped and was down to 14.0 per 100,000 in the period 2010 to 2012, reported in Cancer in Thailand volume VIII (Imsamran et al., 2015).

Screening is considered an effective preventive intervention for cervical cancer. Organized countrywide cytological cervical cancer screening in many countries has demonstrated reduction in both incidence and mortality (Mahlck et al., 1994; Dickinson et al., 2012). In Sweden decrease has been documented for squamous cell carcinomas but not adenocarcinomas (Gunnel et al., 2007). Other results indicated that Pap smear screening has decreased the incidence and mortality of squamous cell carcinoma in many countries with more limited efficacy regarding the adenocarcinoma incidence (Mathew and George, 2009).

For cervical cancer screening in Thailand, a Pap smear program was launched in particular hospitals in 1960s (Yothasamut et al., 2010). Visual inspection with acetic acid (VIA) was recommended for opportunistic cervical screening before 2000. In 2002, the Ministry of Public Health (MoPH) and the National Health Security Office (NHSO) introduced a countrywide systematic screening program for women aged 35-60 years with a 5-year interval under a national universal coverage scheme (Sriamporn et al., 2006). The goal was to reduce the cervical cancer incidence by 50% every 5 years, with 2 phases envisaged for 2005-2009 and 2010-2014. The detection methods were the Pap smear and, in some places, VIA, based on experience and resources (Srivatanakul, 2004).

Khon Kaen province located in the northeastern of Thailand covers an area 10,886 square kilometers with a population of 1.7 million (Imsamran et al., 2015). The incidence of cervical cancer was second highest in the country during the period of 1988 to 1991. Although the estimated ASR of cervical cancer in the province demonstrated continuous reduction down to 10.4 per 100,000 in 2010 to 2012, it remained the third most common cancer type in women of Khon Kaen. The organized cervical cancer screening program according to the universal coverage (UC) scheme was implemented in 2004, with the Pap smear test as the method. This study aimed to determine change in cervical cancer incidence rates in the province from 1990–2014 and to predict future trends until 2029.

## Materials and Methods

*Cancer registration and patient recruitment*

The population of northeastern Thailand at the 2010 census was 18.9 million people of which 9.7 million were females (National Statistical Office, 2012). The population-based cancer registry of Khon Kaen covers 26 districts in northeastern Thailand, with data collected from Srinagarind hospital, Khon Kaen provincial hospital, 3 private hospitals, 24 community hospitals and 1 army hospital (Imsamran et al., 2015). Cervical cancer cases were extracted from the Khon Kaen Population based Cancer Registry from 1990 through 2014 using the International code disease-oncology 3 edition (ICD-O- 3rd) code C53 (WHO, 2017). In addition, information collected from the population-based cancer registry for analyzing age-period-cohort regression models included age and date of diagnosis.

*Population denominators*

Population denominators for calculation of incidence rates were provided by the National Statistical Office for 1990, 2000, and 2010 (National Statistical Office, 1992; 2002; 2012). Population denominators by both sexes for all districts were presented in the censuses. Inter-census populations were estimated using a log-linear function between two consecutive censuses. Populations beyond 2010 were available from the Office of the National Economic and Social Development Board (2013).

*Statistical analysis*

Age-standardized incidence rates (ASRs) were calculated for eighteen different age groups (0-4, 5-9, …, 80-84, and 85+) and twenty-five calendar periods from 1990 to 2014 (1-year intervals). ASRs standardized to the world population proposed by Segi (1960) and later modified by Doll (1976) were estimated for each particular year and plotted to visually illustrate changes over time. 

We analyzed trends in incidence using the Joinpoint Regression Program version 4.4.0.0 (2017) developed by the statistical research and applications branch of the US National Cancer Institute (NCI). Statistically significant change points were obtained by Joinpoint regression. In addition, the rate of change (annual percent change) in each trend segment was calculated using a Monte Carlo permutation method (Kim et al., 2000).

Age-period-cohort (APC) regression models were used to examine effects of age, calendar year, and birth-cohort on cervical cancer incidence. We used the classical method which fits a log-linear model with a Poisson distribution to the observed data using a multiplicative APC model. Two-effect models (age-period and age-cohort) were first chosen and the remaining effect (cohort or period) was then determined for the respective model’s residuals using natural splines to reduce random variation and address the non-identifiability problem of the APC models; therefore, these are referred to as the AP-C and AC-P models. Analysis of APC models was accomplished with the Epi package for R statistical software version 3.4.1 (R Core Team, 2017).

The Norpred package (2017) was adapted to R statistical software and was used to generate projection and graph plots fitting an APC model. Then world-standardized incidence rates for eighteen age groups (from 0-4 to 85+, 5-year intervals) and 5-year interval periods (1990-1994, …, 2010-2014) were employed. Three independent methods were employed to project the incidence rates of cervical cancer in Khon Kaen province; Joinpoint, age-period cohort, and Nordpred projection.

The research was approved by the Khon Kaen University Ethics Committee for Human Research (reference no.HE601184).

## Results

The total number of cervical cancer in Khon Kaen was 3,258 cases from 1990 to 2014. The mean age was 51.5 (SD, 12.4) years. According to time period, from 1990 to 1994, 1995 to 1999, 2000 to 2004, 2005 to 2009, and 2010 to 2014, the age group with the largest number of cases were between 45 and 49, 40 and 44, 40 and 44, 45 and 49, 45 and 49 years of age, respectively. The histology of primary evidence was mostly used as the basis of diagnosis. As for the stage of disease, the most common stage of disease was ‘Stage III’ in 1990-1994, 1995-1999, 2000-2004, and 2005-2009; however, the most common stage of disease was ‘Unknown staging’ between 2010 and 2014. The most histological type was squamous cell carcinoma. For the histological grading, the common histology was unknown (Table 1).

**Table 1:** Characteristics of Cervical Cancer in Khon Kaen by 5-Year Periods

Characteristics	1990 -1994		1995 - 1999		2000 - 2004		2005 - 2009		2010 - 2014	
	n	%	n	%	n	%	n	%	n	%
Age at diagnosis										
20 - 24	8	1.2	2	0.3	1	0.1	2	0.3	2	0.3
25 - 29	12	1.9	4	0.7	10	1.4	6	0.9	12	2.0
30 - 34	41	6.3	30	4.8	42	6.0	30	4.4	25	4.2
35 - 39	71	10.9	55	8.8	74	10.6	77	11.2	52	8.7
40 - 44	92	14.2	103	16.5	109	15.5	108	15.7	79	13.2
45 - 49	103	15.9	93	14.9	100	14.3	115	16.7	85	14.2
50 - 54	96	14.8	90	14.5	101	14.4	99	14.4	83	13.9
55 - 59	82	12.6	91	14.6	86	12.3	80	11.6	76	12.7
60 - 64	65	10	63	10.1	67	9.6	62	9.0	60	10.1
65 - 69	42	6.5	42	6.7	46	6.6	51	7.4	40	6.7
70 - 74	24	3.7	21	3.4	36	5.1	22	3.2	41	6.9
75 - 79	8	1.2	14	2.3	19	2.7	23	3.3	26	4.4
80 - 84	4	0.6	9	1.4	7	1.0	10	1.5	10	1.7
85+	1	0.2	6	1.0	3	0.4	3	0.4	6	1.0
Mean(SD) = 51.5(12.4)	49.8 (12.0)		51.9 (12.2)		51.3 (12.4)		51.3 (12.2)		53.1 (13.3)	
Median (IQ) = 50(42:60)	49 (41:58)		51 (43:59)		50 (42:60)		50 (42:59)		52 (43:62)	
Basis of diagnosis										
Death Certificate Only	23	3.5	7	1.1	5	0.7	2	0.3	2	0.3
History and Physical exam	125	19.3	107	17.2	86	12.3	42	6.1	33	5.5
Endoscopy and Radiology	8	1.2	10	1.6	5	0.7	6	0.9	12	2.0
Surgery and Autopsy (no history)	4	0.6	7	1.1	9	1.3	3	0.4	4	0.7
Specific Biochem / Immuneno test	-	-	-	-	-	-	-	-	-	-
Cytology of Hematology	14	2.2	16	2.6	24	3.4	7	1	3	0.5
Histology of Metastasis	-	-	-	-	-	-	-	-	-	-
Histology of Primary	475	73.2	476	76.4	572	81.6	628	91.3	543	91
Autopsy with histology	-	-	-	-	-	-	-	-	-	-
Stage at diagnosis										
Stage I	102	15.7	90	14.4	136	19.4	192	27.9	152	25.4
Stage II	123	19.0	155	24.9	161	23	156	22.7	133	22.3
Stage III	196	30.2	168	27.0	184	26.2	194	28.2	121	20.3
Stage IV	41	6.3	43	6.9	44	6.3	51	7.4	29	4.9
Unknown	187	28.8	167	26.8	176	25.1	95	13.8	162	27.1
Histological type										
Squamous cell carcinoma	404	62.2	406	65.2	458	65.3	482	70.0	439	73.5
Adenocarcinoma	68	10.5	63	10.1	109	15.6	123	17.9	90	15.1
Unspecified and Other	177	27.3	154	24.7	134	19.1	83	12.1	68	11.4
Histological grading										
Well-differentiated	-	-	12	1.9	40	5.7	78	11.3	45	7.6
Moderately-differentiated	3	0.4	16	2.6	37	5.3	169	24.6	213	35.7
Poorly-differentiated	1	0.2	7	1.1	13	1.9	30	4.4	113	18.9
Undifferentiated	-	-	-	-	1	0.1	-	-	2	0.3
Unknown	645	99.4	588	94.4	610	87	411	59.7	224	37.5

**Table 2:** Joinpoint Analysis Identifies Three Trends at 1990-2005, 2005-2008, and 2008-2014

Trend 1		Trend 2		Trend 3		AAPC
Period	APC (95%CI)	Period	APC (95%CI)	Period	APC (95%CI)	1990 - 2014
1990-2005	-2.8 (-4.5, -1.1)*	2005-2008	3.6 (-31.6, 56.8)	2008-2014	-8.0 (-14.5, -1.1)*	-3.4 (-8.3, 1.8)

*, Significant (P-value <0.05)

**Table 3:** Joinpoint Analysis Identifies Three Trends of Cervical Cancer Separated by Histological Type at 1990-2005, 2005-2008, and 2008-2014

Histological type	Trend 1		Trend 2		Trend 3		AAPC
	Period	APC (95%CI)	Period	APC (95%CI)	Period	APC (95%CI)	1990 - 2014
Squamous cell carcinoma	1990-2005	-2.4 (-4.6, -0.1)*	2005-2008	7.5 (-34.5, 76.4)	2008-2014	-9.6 (-17.5, -1.0)*	-3.1 (-9.0, 3.2)
Adenocarcinoma	1990-2014	0.4 (-1.3, 2.2)	-	-	-	-	0.4 (-1.3, 2.2)
Unspecified and Other	1990-2014	-6.8 (-8.5, -5.1)*	-	-	-	-	-6.8 (-8.5, -5.1)*

*, Significant (P-value <0.05)

**Figure 1:**
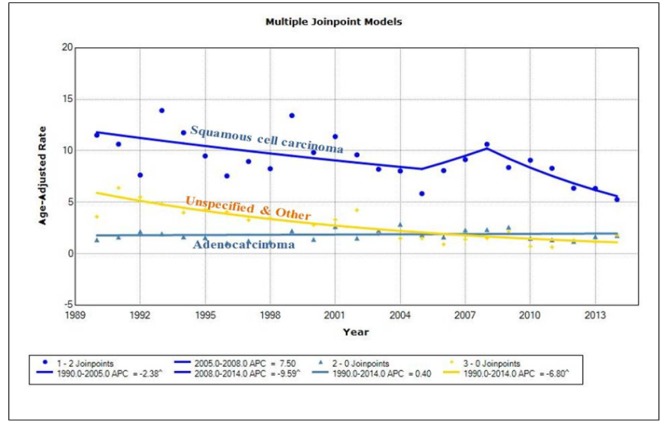
Trends in Incidence of Cervical Cancer Separated by Histological Type in Khon Kaen from 1990 to 2014

**Figure 2:**
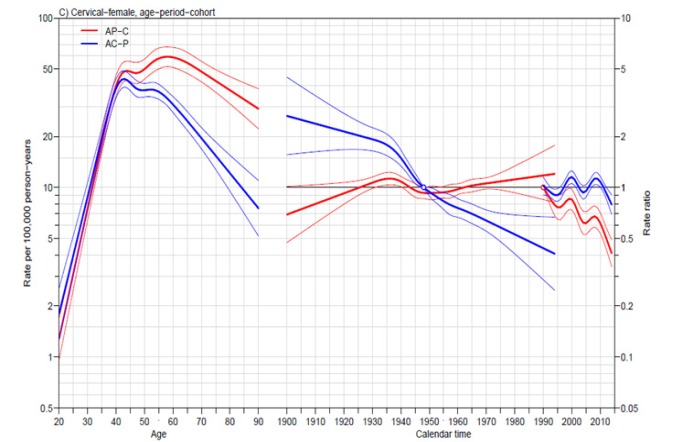
Trends in Incidence of Cervical Cancer by Age-Period-Cohort Model

**Figure 3:**
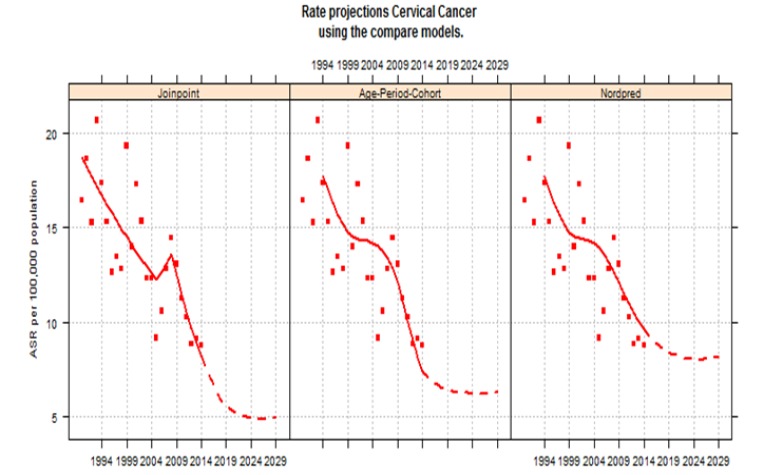
Future Trend of Cervical Cancer in Khon Kaen Using the Compare Model

Between 1990 and 2014, a total of 3,258 cases were diagnosed with ICD-O code C53 (invasive cervical cancer). Before the year 2005, the annual percentage change (APC) varied widely, with marked outliers in 1993 and 1999. The average decrease computed with Joinpoint software was -2.8% (95% CI; -4.5 to -1.1) per year. After 2005, a rise in ASR was noted until 2008, with a subsequent clear drop after 2008 which continued at -8.0% (95% CI; -14.5 to -1.1) per year on average (Table 2).

An annual percentage change (APC) for squamous cell carcinoma was -2.4% (95% CI; -4.6 to -0.1) per year on average from 1990 to 2005, followed by a rise until 2008, then a continuous fall in ASR with an APC of -9.6% (95% CI; -17.5 to -1.0) per year on average. For adenocarcinomas, there was no apparent change from 1990 to 2014. For other & unspecified lesions, the APC from 1990 to 2014 was -6.8% (95% CI; -8.5 to -5.1) per year on average (Table 3 and Figure 1).

Regarding the influence of age, year at diagnosis and year of birth, both AC-P and AP-C models showed incidence to peak at around 40 to 60 years of age. The incidence rate of patients diagnosed in 2014 was 0.4 times that of their counterparts were diagnosed in 1990 (AP-C model). Moreover, the incidence rate for patients born in 1994 was 0.4 times that of those born in 1948 (AC-P model) (Figure 2).

Projection using the geometric cut trend indicated that incidence rates would be expected to continuously decrease in the future till 2029, Joinpoint regression demonstrating a drop from 8.2 cases per 100,000 populations in 2014 to 5.0 in 2029. The age-period-cohort model showed ASR reduction from 7.4 in 2014 to 6.3 in 2029. Furthermore, NordPred decrease from 9.6 in 2014 to 8.2 in 2029 (Figure 3).

## Discussion

The current study showed that the incidence of cervical cancer has been decreasing by -3.4% per year (AAPC). Before the year 2005, the annual percentage change (APC) varied widely, with marked outliers in 1993 and 1999. The average decrease was -2.8% (95% CI; -4.5 to -1.1) per year. After 2005, a rise in ASR was noted until 2008, with a subsequent clear drop after 2008 which continued at -8.0% (95% CI; -14.5 to -1.1) per year. This is consistent with the ASRs of cervical cancer as report by the Thailand cancer registry (Vatanasapt et al., 1993; Deerasamee et al., 1999; Sriplung et al., 2003; Khuhaprema et al., 2007; 2010; 2012; 2013; Imsamran et al., 2015). Similar results were reported in other studies. A study of trends in incidence of cervical cancer in Songkhla, southern Thailand found that the APC was increasing at 1.5% per year before 2000. The drop in ASR after the year 2000 was obvious and continuous with APC of -4.7% per year (Sriplung et al., 2014). Furthermore, a study of national and subnational population-based incidence of cancer in Thailand found that the average increase of cervical cancer was 9.9% per year before 1995. After the year 1995, a decrease in ASR was noted until 2008 and largely decreased in last 5 years (2008-2012) with APC of -12.5.0% per year in the north. In the central, APC was increasing at 3.3% per year before 2006 and largely decreased in last 7 years (2006-2012) with APC of -10.2% per year. In the south, the APC was increasing at 2.1% per year before 2000. The drop in ASR after the year 2000 was obvious and continuous with APC of -5.0% per year (Virani et al., 2017). We have hypothesized that the decline in the incidence of cervical cancer might be the result of the organized screening program. After the organized screening program in Thailand was set up in 2002 by the Ministry of Public Health (MoPH) and the National Health Security Office (NHSO) offered Pap smear screening to the entire female population between 35 and 60 years old with a 5-years interval under the national universal coverage scheme (Yothasamut et al., 2010), trends in incidence demonstrated obvious decrease between 2008 and 2014. Nevertheless, the present study showed that Khon Kaen had decreased until 2005 before the cervical cancer screening tests was covered by national Universal Coverage scheme in 2004 (Table 2, 3 and Figure 1). Therefore, the decline in the incidence of cervical cancer before 2004 might be the result of opportunistic cervical cancer screenings. Before the year 2004, there was mass screening for cervical cancer in Khon Kaen. This screening began in 1986 by the faculty of Medicine, Khon Kaen University in collaboration with others health organization in the province. The short period campaign was in October 13-17, 1986 with the 20 districts hospitals supplying Pap smear in women age 14-94 years old. Total 9,508 women from the total of 763,671 women joined the activities with the results of genital infection in 4,138 women (43.5%) and abnormal cervical cell in 140 women (1.3%). The long term campaign began after that week until one year later, the total 6,835 women attended with 186 cases (2.7%) of genital infection and 63 cases (0.9%) of abnormal Pap smear reported. All abnormal cases were referred to Srinagarind hospital, Khon Kaen University for further investigation and treatment (Vatanasapt et al., 1992). In addition, a total of 16,648 women in Khon Kaen province had been enrolled in the Khon Kaen Cohort Study (KKCS) between 1990 and 2001. Pap smears were obtained from 10,954, and cervical cells from 10,073, participants having abnormal Pap test, results being advised to seek treatment (Sriamporn et al., 2005). The effects of this effort can be seen in the reduction of early cervical cancer incidence in this study. Thus, trend in incidence of cervical cancer in Khon Kaen are different from other regions in Thailand. 

Both AC-P and AP-C models show the same fact that the incidence of cervical cancer in Khon Kaen peaks around 40 to 60 years (Figure 2). The incidence rate of the patients who were diagnosed in 2014 was 0.4 times when compared with the patients who were diagnosed in 1990 (AP-C model). Moreover, the incidence rate of the patients who were born in 1994 was 0.4 times when compared with the patients who were born in 1948 (AC-P model). The consistency of the AP-C and AC-P from age-period-cohort model showed that the change was influenced by the period effect (year at diagnosis) and cohort effect (year of birth). The evidences showed the success of the opportunistic and the countrywide systematic screening programs by using Pap smear screening.

The projection showed the incidence rates would be continuously decreasing in the future till 2029 (Figure 3). This is consistent with the reported incidence in a previous study which studied the trend in incidence of five cancers and prediction of future trends in each region of Thailand. The study found that the incidence rates of other regions (north, central, south) would be continuously decreasing in the future until 2030 (Virani et al., 2017). Thus, the projection is ensured when there is no major change affecting the screening scheme in the near future if the screening is still continuing indefinitely. It could be concluded that the decreasing of trend in cervical cancer incidence in Khon Kaen continues to decline into the future and the declining trend in cervical cancer incidences must be maintained to achieve the predicted goal of ASRs below 10 or even 5 per 100,000 women by 2029.

The major limitation in this study was the fact that the definite timeline of opportunistic cervical cancer screening campaigns of previous times in Khon Kaen before 2002 could not be evidently identified. Thus, we missed some information for elucidating the trend. However, the essential changes in cervical cancer incidence and trends in this study appear to be consistent with the overall health care system and timeline of cervical cancer screening campaigns in the country; therefore, our conclusion should be correct.

The best way to understand the nature of disease in a population is analysis of population-based cancer registry data. This study has shown decreasing trend in incidence of cervical cancer in Khon Kaen, a province in Northeastern Thailand from 1990 to 2014 and would be decreased continuously to 2029. The decreasing of trend in cervical cancer incidence is due to the countrywide systematic screening programs, which is the most economical and routine to do normally in the public health system. There is no need to increase the country’s expenditure on HPV vaccines. Consequently, the screening program should be maintained and improved continuously.
